# Bridging the Gap in Diabetes and Fracture Care for Diverse Populations

**DOI:** 10.1089/whr.2024.0200

**Published:** 2025-03-07

**Authors:** Nicklas H. Rasmussen

**Affiliations:** Steno Diabetes Center North Denmark, Aalborg University Hospital, Aalborg, Denmark.

The intersection of diabetes and bone health presents a critical, yet often overlooked, challenge in modern medicine. Type 2 diabetes mellitus (T2D), a condition affecting millions globally, is associated with an increased risk of falls and fractures, disproportionately impacting women.^1,2^ These findings resonate with the results presented in the article *Ahmed F, Miller K, Beganovic M, Siddiqui A, Smith C. A Sex Comparison of Fall and Fracture Occurrence in the Elderly Diabetic Population: A Quantitative Study. Women’s Health Reports 2025; volume 6, issue 1.*.

The article highlights significant sex disparities in fracture risk among people with T2D, where women are disproportionately affected despite similar fall rates between sexes. This suggests intrinsic factors—beyond the mechanical impact of falls—play a crucial role in diabetic bone fragility. Such disparities demand a nuanced understanding of the pathophysiology of bone health in T2D and proactive measures to mitigate these risks.

## Understanding the Biology of Bone Fragility in T2D

The mechanisms underlying bone fragility in T2D are multifaceted.^3^ Hyperglycemia leads to the accumulation of advanced glycation end-products (AGEs) in bone collagen, impairing bone quality and structural integrity.^4^ Moreover, T2D-related insulin resistance compromises osteoblast function, reducing bone formation.^5^ Physiologically, postmenopausal estrogen deficiency accelerates bone loss, a process compounded by diabetes-induced reductions in bone quality. These biological factors, compounded by sociocultural determinants such as access to health care, socioeconomic status, and cultural practices affecting diet and physical activity, underscore why diabetic women—especially postmenopausal women—face heightened fracture risks.^6^

## Sex-Specific Risk and Clinical Implications

Ahmed et al.’s study leverages a retrospective cohort design, allowing for a robust analysis of sex-specific fracture risks in a diverse population. Their findings highlight a significant disparity, with women experiencing a markedly higher fracture rate (50%) compared to men (26%), despite comparable fall frequencies. However, the inherent limitations of retrospective designs, such as potential information bias, should be acknowledged. Although this divergence points to underlying biological and sociocultural factors that amplify fracture risk among women with T2D. Historically, research on bone health and diabetes has predominantly focused on Caucasian cohorts.^7^ Ahmed et al.’s inclusion of a population where 72% are Black addresses this critical gap, providing valuable insights into fracture risks among underrepresented groups. This demographic focus enhances the generalizability of findings, particularly for health care strategies targeting underserved populations. Ahmed et al.’s inclusion of a diverse population underscores the necessity of expanding research frameworks to account for racial and ethnic variability in fracture risk factors, such as access to health care, socioeconomic status, and cultural practices affecting nutrition and physical activity.

Furthermore, behavioral and environmental factors also play a critical role. Women are more likely to adopt protective behaviors postfracture, such as reducing physical activity to avoid future falls. While these adaptations may lower fall risk, they can inadvertently lead to further bone demineralization and muscle atrophy, creating a feedback loop that increases long-term fracture susceptibility. Addressing these behavioral patterns through targeted education and support programs is essential to breaking this cycle.

As emphasized in previous research by Vestergaard and van der Bergh, fracture risk in T2D extends beyond traditional osteoporosis definitions, necessitating more comprehensive diagnostic tools.^8,9^ Advanced imaging methods, including trabecular bone score, high-resolution peripheral quantitative computed tomography, AGE skin readers, and bone biomarkers, may offer a more detailed insight into bone quality within this population.^10–12^

## Preventive Strategies

Effective strategies to mitigate fracture risks in diabetic people include early and routine screening for osteoporosis, particularly among postmenopausal women with long-standing diabetes.^13–15^ Interventions should also address glycemic control, as poor glucose management exacerbates bone fragility.^16,17^ Pharmacological therapies, such as bisphosphonates, selective estrogen receptor modulators, or even newer anabolic therapies, should be carefully considered and tailored to individual risk profiles.^18^ Lifestyle modifications are equally crucial. Weight-bearing exercises and strength training can enhance bone density and improve balance, reducing fall risk.^19^ Adequate calcium and vitamin D intake must also be ensured. Notably, culturally sensitive education and community-based interventions are vital to address disparities in fracture risks among diverse populations.^6^ Culturally sensitive education and community-based programs are crucial for addressing disparities in fracture prevention and promoting adherence to comprehensive diabetes management plans. An overview of a multifaceted approach:

1. Screening and Diagnosis: Early and routine osteoporosis screening should be prioritized for postmenopausal women and people with long-standing diabetes. Leveraging advanced imaging tools can improve risk stratification and early detection.
2. Pharmacological Interventions: Treatment plans must include evidence-based pharmacotherapies, such as bisphosphonates, selective estrogen receptor modulators, or newer anabolic agents, tailored to the patient’s fracture risk profile and glycemic control requirements.
3. Lifestyle Modifications: Exercise regimens focused on weight-bearing and resistance training improve bone strength and balance, reducing fall risk. Ensuring adequate intake of calcium and vitamin D remains foundational.
4. Culturally Sensitive Education: Community-based programs tailored to diverse populations can address disparities by promoting awareness of fracture prevention strategies and adherence to diabetes management plans.

## Contextualizing the Findings in Broader Research

Research from Denmark supports the findings of Ahmed et al., highlighting elevated fracture risks among people with T2D, particularly due to diabetes-related comorbidities.^20^ These studies emphasize the compounded risks arising from impaired postural control and musculoskeletal fragility.^21^ Such data underline the need for integrating musculoskeletal assessments and personalized interventions into standard diabetes care. But also, identifying subtypes of T2D, as not all are equally at risk of falls and fractures.^22^ These approaches aim to preemptively mitigate vulnerabilities and improve long-term outcomes in people with T2D.^23^

## The Need for Interdisciplinary Collaboration

The referenced study and related research collectively point to the need for interdisciplinary care models that integrate endocrinology, orthopedics, geriatrics, and primary care. Such collaboration can facilitate holistic care models that address the multifactorial nature of fracture risk in T2D, ensuring personalized and comprehensive patient care.

## Conclusion

The intersection of T2D and bone health constitutes a critical challenge in clinical and research domains, necessitating targeted and interdisciplinary solutions. Studies such as *Ahmed F, Miller K, Beganovic M, Siddiqui A, Smith C. A Sex Comparison of Fall and Fracture Occurrence in the Elderly Diabetic Population: A Quantitative Study. Women’s Health Reports 2025; volume 6, issue 1,*. shine a light on disparities that must inform future prevention strategies. By advancing our understanding of the underlying mechanisms and leveraging interdisciplinary expertise, we can reduce the burden of fractures and improve health outcomes for all people with diabetes.

## Abbreviations Used

AGE: Advanced Glycation End-products
DXA: Dual-energy X-ray Absorptiometry
HR-pQCT: High-Resolution peripheral Quantitative Computed Tomography
T2D: Type 2 Diabetes Mellitus
TBS: Trabecular Bone Score

## References

[1] Rasmussen NH, Dal J. Falls and fractures in diabetes—more than bone fragility. Curr Osteoporos Rep 2019;17(3):147–156. 30915638 10.1007/s11914-019-00513-1

[2] Guirguis-Blake JM, Michael YL, Perdue LA, et al. Interventions to prevent falls in older adults: Updated evidence report and systematic review for the US preventive services task force. JAMA—JAMA 2018;319(16):1705–1716.10.1001/jama.2017.2196229710140

[3] Van Hulten V, Rasmussen N, Driessen JHM, et al. Fracture patterns in type 1 and type 2 diabetes mellitus: A narrative review of recent literature. Curr Osteoporos Rep 2021;19(6):644–655. 34931295 10.1007/s11914-021-00715-6 PMC8716348

[4] Hygum K, Starup-Linde J, Harsløf T, et al. Diabetes mellitus, a state of low bone turnover-a systematic review and meta-analysis. Eur J Endocrinol 2017;176(3):R137–R157. 28049653 10.1530/EJE-16-0652

[5] Linde JS, Hygum K, Langdahl BL. Skeletal fragility in type 2 diabetes mellitus. Endocrinol Metab 2018;33(3):339–351. 10.3803/EnM.2018.33.3.339 PMC6145952 30229573

[6] Eastell R, Rosen CJ, Black DM, et al. Pharmacological management of osteoporosis in postmenopausal women: An endocrine society* clinical practice guideline. J Clin Endocrinol Metab 2019;104(5):1595–1622. 30907953 10.1210/jc.2019-00221

[7] Compston J. Type 2 diabetes mellitus and bone. J Intern Med 2018;283(2):140–153. 29265670 10.1111/joim.12725

[8] Starup-Linde J, Vestergaard P. Diabetes and osteoporosis: Cause for concern? Eur J Endocrinol 2015;173(3):R93–R99. 26243638 10.1530/EJE-15-0155

[9] Eller-Vainicher C, Cairoli E, Grassi G, et al. Pathophysiology and management of type 2 diabetes mellitus bone fragility. J Diabetes Res 2020;2020:7608964; doi: 10.1155/2020/7608964 32566682 PMC7262667

[10] Starup-Linde J, Gregersen S, Vestergaard P. Associations with fracture in patients with diabetes: A nested case-control study. BMJ Open 2016;6(2):e009686; doi: 10.1136/bmjopen-2015-009686 PMC4762072 26873048

[11] Lekkala S, Sacher SE, Taylor EA, et al. Increased advanced glycation endproducts, stiffness, and hardness in iliac crest bone from postmenopausal women with type 2 diabetes mellitus on insulin. J Bone Miner Res 2023;38(2):261–277. 36478472 10.1002/jbmr.4757 PMC9898222

[12] Farr JN, Drake MT, Amin S, et al. *In vivo* assessment of bone quality in postmenopausal women with type 2 diabetes. J Bone Miner Res 2014;29(4):787–795. 24123088 10.1002/jbmr.2106 PMC3961509

[13] Rasmussen NH, Vestergaard P. Diabetes and osteoporosis - Treating two entities: A challenge or cause for concern? Best Pract Res Clin Rheumatol 2022;36(3):101779; doi: 10.1016/J.BERH.2022.101779 36154803

[14] Wettasinghe AH, Dissanayake DWN, Allet L, et al. Falls in older people with diabetes: Identification of simple screening measures and explanatory risk factors. Prim Care Diabetes 2020;14(6):723–728. 32473990 10.1016/j.pcd.2020.05.006

[15] Rasmussen NH, Thomsen RW, Rasmussen HH, et al. Validity of diagnostic coding for undernutrition in hospitals. Clin Nutr 2016;35(2):491–495. 25892602 10.1016/j.clnu.2015.03.017

[16] Schwartz AV, Vittinghoff E, Sellmeyer DE, et al; Health, Aging, and Body Composition Study. Diabetes-related complications, glycemic control, and falls in older adults. Diabetes Care 2008;31(3):391–396. 18056893 10.2337/dc07-1152 PMC2288549

[17] Tancredi M, Rosengren A, Svensson A-M, et al. Excess mortality among persons with type 2 diabetes. N Engl J Med 2015;373(18):1720–1732. 26510021 10.1056/NEJMoa1504347

[18] Cheng CH, Chen LR, Chen KH. Osteoporosis due to hormone imbalance: An overview of the effects of estrogen deficiency and glucocorticoid overuse on bone turnover. Int J Mol Sci 2022;23(3); doi: 10.3390/IJMS23031376 PMC8836058 35163300

[19] Power GM, Tobias JH, Frayling TM, et al. Age-specific effects of weight-based body size on fracture risk in later life: A lifecourse Mendelian randomisation study. Eur J Epidemiol 2023;38(7):795–807. 37133737 10.1007/s10654-023-00986-6 PMC10276076

[20] Rasmussen NH, Dal J, den Bergh J, et al. Increased risk of falls, fall-related injuries and fractures in people with type 1 and type 2 diabetes—a nationwide cohort study. Curr Drug Saf 2021;16(1):52–61. 32900349 10.2174/1574886315666200908110058

[21] Rasmussen NH-H, Dal J, Jensen MH, et al. Impaired postural control in diabetes-a predictor of falls? Arch Osteoporos 2022;18(1):6. 36482222 10.1007/s11657-022-01188-5

[22] Ahlqvist E, Prasad RB, Groop L. Subtypes of type 2 diabetes determined from clinical parameters. Diabetes 2020;69(10):2086–2093. 32843567 10.2337/dbi20-0001

[23] Cichosz SL, Rasmussen NH, Vestergaard P, et al. Is predicted body-composition and relative fat mass an alternative to body-mass index and waist circumference for disease risk estimation? Diabetes Metab Syndr 2022;16(9):102590; doi: 10.1016/J.DSX.2022.102590 35986982

